# Lignin-Based Nanoparticles: A Review on Their Preparations and Applications

**DOI:** 10.3390/polym12112471

**Published:** 2020-10-25

**Authors:** Qianqian Tang, Yong Qian, Dongjie Yang, Xueqing Qiu, Yanlin Qin, Mingsong Zhou

**Affiliations:** 1 College of Chemistry and Chemical Engineering, Henan Key Laboratory of Function-Oriented Porous Materials, Luoyang Normal University, Luoyang 471934, China; lhltqq1987@163.com; 2 School of Chemistry and Chemical Engineering, State Key Laboratory of Pulp and Paper Engineering, South China University of Technology, Guangzhou 510640, China; ceyqian@scut.edu.cn (Y.Q.); cedjyang@scut.edu.cn (D.Y.); 3 School of Chemical Engineering and Light Industry, Guangdong University of Technology, Guangzhou 510006, China; xueqingqiu66@163.com

**Keywords:** lignin, lignin-based nanoparticles, preparations, applications

## Abstract

Lignin is the most abundant by-product from the pulp and paper industry as well as the second most abundant natural renewable biopolymer after cellulose on earth. In recent years, transforming unordered and complicated lignin into ordered and uniform nanoparticles has attracted wide attention due to their excellent properties such as controlled structures and sizes, better miscibility with polymers, and improved antioxidant activity. In this review, we first introduce five important technical lignin from different sources and then provide a comprehensive overview of the recent progress of preparation techniques which are involved in the fabrication of various lignin-based nanoparticles and their industrial applications in different fields such as drug delivery carriers, UV absorbents, hybrid nanocomposites, antioxidant agents, antibacterial agents, adsorbents for heavy metal ions and dyes, and anticorrosion nanofillers.

## 1. Introduction

As a green, safe, low-cost, and sustainable natural renewable resource, lignocellulose is considered an excellent potential alternative to traditional petrochemical resources for a variety of applications [1]. In recent years, producing bioenergy and high-value green chemicals from lignocellulose has received worldwide attention due to the increasing resource, environmental, economic, and health issues [2,3,4]. Lignocellulose is derived from plant and wood, which mainly consists of cellulose, hemicellulose, and lignin [5]. Currently, the researches on the applicability of carbohydrates are mostly focused on cellulose and hemicellulose systems. However, lignin, as the second most abundant biopolymer next to cellulose on earth, is underutilized and its potential application value has not yet been well exploited [6,7,8].

**Figure 1:**
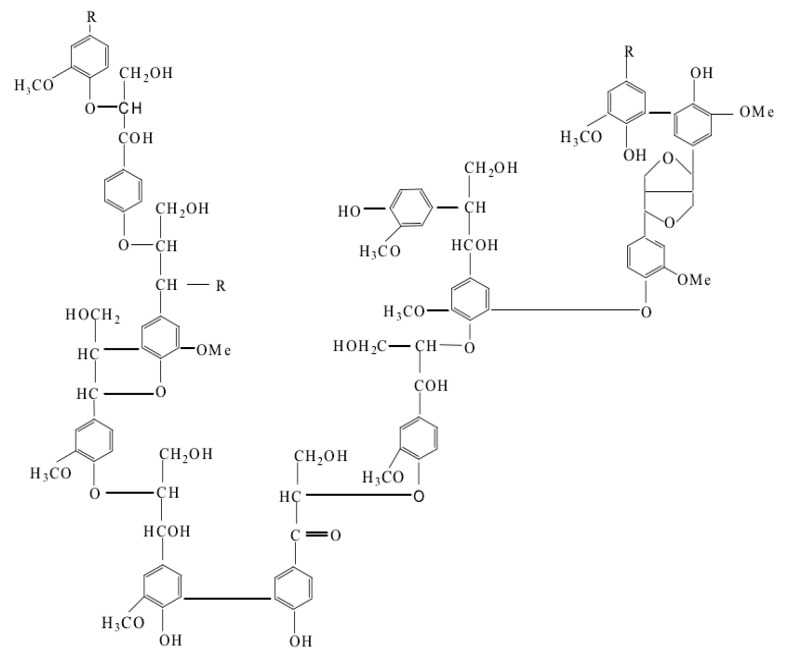
The possible structural representation of lignin [24].

Lignin is copious and also the largest reservoir of aromatic polymer on earth, which plays an important role in plants, including transporting water and minerals, providing mechanical supports, and protecting plants or wood from chemical or microbial attacks [9,10,11]. The molecular structure of lignin is highly dependent on the wood locations, species, and especially the extraction processes [10,12,13,14,15,16]. Different types of lignin contain different functional groups and show different molecular weight and elemental composition [17]. Therefore, the structure of lignin is extremely complicated and difficult to determine. However, it is generally accepted that lignin is a highly branched, amorphous, and three-dimensional network biomacromolecule, which consists of three basic phenylpropane monomers: guaiacyl, syringyl, and *p*-hydrophenyl [5] linked together by a number of bonds including several types of carbon-carbon and ether linkage [18,19,20,21,22,23]. Figure 1 gives the possible structural representation of lignin molecules [24]. From Figure 1, there are a large number of active functional groups in lignin, such as aliphatic and phenolic hydroxyl groups, carbonyl groups, methoxy groups, and phenyl groups, which are important active sites for further chemical modifications of lignin by sulfonation, oxidation, graft copolymerization, or hydroxymethylation reactions, etc. [25,26,27,28,29,30]. The obtained modified lignin products could be utilized in different industrial fields [5,31,32,33,34,35].

Besides the natural abundance, lignin is also present as a major byproduct of the pulp and paper industry [36]. In the paper pulping process, most of the lignin is removed and discharged in the form of spent liquors. Every year about 50 million tons of lignin is generated from the pulp and paper industry [37,38]. However, the majority is discarded as waste or burnt to recover heat and electricity [39,40,41], causing serious environmental pollution and resource waste. Only approximately 2% of the produced lignin is isolated and effectively used for various products [9], including industrial dispersants [42,43,44,45,46,47,48,49,50], cleaning agents [51], and dopants for conductive polymers [36]. Theoretically, lignin should be a remarkable feedstock for different materials and could be widely used in different fields due to its aromaticity, various reactive functional groups, excellent ultraviolet and oxidation resistance, high thermal stability, nontoxicity, biodegradability, renewability, and low costs [22,24,52,53,54,55]. However, only a very small portion of lignin is utilized. This low utilization percentage mainly results from the highly complex and changeable molecular structure, poor miscibility with a host polymer matrix, and high polydispersity of lignin [56,57,58]. Additionally, the complexity of its isolation, purification, chemical modifications, and structural characterization also inhibits its high value-added applications [1]. In any case, finding new and high value-added applications of lignin recovered from pulping waste liquor is imperative, which has both economic and environmental benefits. In light of the excellent properties and extensive applications of nanomaterials in various fields, it has been considered to prepare nanospheres from lignin, which provides the possibility to utilize lignin-based products in high value-added industrial fields, such as UV-blocking additives for thermoplastics, drug delivery, and Pickering emulsions [7].

In this review, we provide a comprehensive overview of the preparation of lignin-based nanospheres and their applications in different commercial fields. The aim of this article is to attract considerable attention from the target researches towards developing high value-added industrial applications of lignin-based products.

## 2. Main Types of Lignin

Lignin could be extracted or separated from lignocellulosic biomass. Different extraction or separation processes would result in different physical and chemical properties of products [17,56], such as surface properties, solubility, molecular weight, and functional groups [59], which greatly affect the preparation, performance, and even application of nanoparticles. At present, there are mainly five pretreatment processes employed to separate lignin, which are sulfite, kraft, organosolv, soda, and enzymatic hydrolysis processes [1,10]. Ultimately, five types of technical lignin products are obtained and summed up in Table 1. Among them, lignosulfonate, kraft, and soda lignin are produced at commercial scales through LignoForce, LignoBoost, and Howard methods, etc. Therefore, they could be easily obtained in large quantities. However, organosolv and enzymatic hydrolysis lignin could only be produced at small laboratory scales due to the lack of commercial production methods.

### 2.1. Lignosulfonate

Lignosulfonate is obtained from the sulfite pulping process, which involves the reactions between lignin and sulfite salts or sulfurous acids with sodium, magnesium, or calcium as counter ions [56,60] at 120–180 °C under either pH-neutral, acidic or alkaline conditions for 1–5 h. In this lignin, sulfonic groups are introduced. Together with other hydrophilic groups such as carboxyl and phenolic hydroxyl groups, the aqueous solubility of lignosulfonate is greatly improved [61]. Besides, lignosulfonate also contains hydrophobic groups such as aliphatic and aromatic groups and is therefore considered to be an anionic surfactant. Due to the favorable solubility and certain surface activity, lignosulfonate has been widely used in different commercial fields, such as water-coal-slurry dispersants, dye dispersants, concrete water reducers, oil-well dispersants, and pesticide dispersants [7], owning the advantages of wide sources of raw material, low costs, environmental friendliness, and safety. However, lignosulfonate also presents some disadvantages, such as the structural change of lignin due to the formation of new C-C bonds, and the low purity after isolation with high contents of ash and other impurities [5].

### 2.2. Kraft Lignin

Contrary to lignosulfonate, kraft lignin is obtained from the kraft pulping process, which accounts for the highest proportion (approximately 85%) of industrial lignin [55,62]. The conventional kraft pulping process is carried out under high pH values and high temperatures. First, lignin is pretreated with sodium hydroxide and sodium sulfide at temperatures of about 170 °C and pH values of 13–14, which would result in the cleavage of ether linkage and introduction of phenolic hydroxyl groups, thus allowing the solubilization of lignin [60,63,64,65]. Then, lignin is separated from the remaining alkaline solutions by acid (such as sulfuric acid) precipitation method, which lowers the pH value to 5–7.5 [5]. In comparison with lignosulfonate, the obtained kraft lignin owns a higher purity due to containing fewer inorganic impurities and carbohydrate residues [41] but contains a smaller amount of sulfur and therefore exhibits a worse water solubility [19]. It could only dissolve under alkaline conditions, which makes kraft lignin an inactive material unless chemically modified [66]. Currently, kraft lignin is mainly employed as a polymer material by blending, as various industrial dispersants after chemical modifications, and as biofuels and biochemicals after chemical pyrolysis or degradation [55].

### 2.3. Organosolv Lignin

Organosolv lignin is obtained from the pulp through treating lignocellulosic biomass using organic solvents as the delignification agents. In this process, the frequently-used organic solvents such as acetone, ethanol, methanol, tetrahydrofurfuryl alcohol, dioxane, ethylene glycol, glycerol, and organic acids, are often mixed with water at temperatures of 170–190 °C [1,64,67,68]. Sometimes, acid or basic catalysts are additionally added to help produce high-quality lignin [64]. This organosolv treatment mainly breaks the α-aryl ether linkages but cleaves the β-aryl ether linkages to a lower amount [69], which causes the formation of new phenolic groups. The resulting lignin has a lower molecular weight and higher purity with lower ash and carbohydrate content than that obtained through other extraction processes [9]. Moreover, it is sulfur-free and basically preserves the native structure of lignin [5]. However, in spite of these advantages, the organosolv lignin is difficult to produce on a large scale due to the extensive corrosion of equipment and the high cost of solvents [1].

### 2.4. Soda Lignin

Soda lignin is produced from the soda pulping process [9], which is often used for treating herbaceous plants such as wheat straw, sugarcane bagasse, kenaf, and flax [1]. During a typical soda process, the lignocellulosic biomass is digested by adding aqueous sodium hydroxide solutions (13–16 wt %) and anthraquinone (as a catalyzer) at temperatures of 140–170 °C [56,60,64]. The molecular weight of the obtained soda lignin varies between 1000 to 3000 Da and the mean weight is around 2400 Da [60,64,70]. Due to the oxidation of aliphatic hydroxyl groups, the soda lignin has a high content of carboxylic acid, which makes it difficult to recover through centrifugation or filtration [56]. However, the soda lignin is sulfur-free and thus has additional advantages for some high value-added applications such as in bioplastics or composites [41].

### 2.5. Enzymatic Hydrolysis Lignin

Enzymatic hydrolysis lignin is obtained from an enzymatic hydrolysis process, in which cellulases and hemicellulases were utilized to degrade cellulose and hemicellulose in biomass, leaving lignin as solid insoluble residues. This whole process has the advantages of eco-friendliness and cost-effectiveness. The obtained lignin products are basically non-sulfur and exhibit very low solubility in either water or some organic solvents. However, they possess a closer structure to native lignin than other technical lignin. Enzymatic hydrolysis lignin generally contains 65–80% of lignin besides other components such as carbohydrates, proteins, and ashes, which has many industrial applications such as dispersants, binders, sorbents, emulsifiers as well as producing various polymeric chemicals [1,10,19].

## 3. Different Preparations Methods for Lignin-Based Nanoparticles

In recent years, different kinds of lignin-based nanoparticles have been prepared based on various types of lignin through different methods such as self-assembly, solvent exchange, acid precipitation, polymerization, ultrasonication, crosslinking, and CO_2_ antisolvent.

### 3.1. Self-assembly Method

Self-assembly is a process in which an ordered or organized structure is generated due to some specific intermolecular noncovalent interactions such as hydrophobic, electrostatic, hydrogen-bonding and Van der Waals interactions in absence of any external directions. This is a frequently-used method to prepare nanoparticles, which we would put emphasis on in this section.

Qian et al. [71] used the self-assembly method to produce the uniform lignin-based colloidal spheres. After acetylation, the alkali lignin (AL) was transformed into acetylated lignin (ACL) and then dissolved into THF. With the gradual addition of water into the ACL/THF solutions, the ACL molecules started to associate to form colloidal spheres through the hydrophobic interaction. After rotary evaporation to remove THF, colloidal spheres with a hydrodynamic radius of 110 nm were successfully obtained. This study gives important enlightenment on how to convert the irregular lignin-based polymers into the ordered colloidal spheres. Qian et al. [72] also reported a novel approach to fabricate lignin reverse micelles (LRMs) via self-assembly. In this method, LRMs were formed by adding cyclohexane into the alkali lignin/dioxane solutions. With the increasing amount of cyclohexane, LRMs were separated from the solutions in the form of precipitation. Deng et al. [73] proposed a simple and feasible method in the formation of hollow lignin azo colloids. They first modified alkali lignin (AL) into the lignin-based azo polymer (AL-azo-H). Then, water was gradually added dropwise into the AL-azo-H/THF solutions. With the further addition of water above 53 vol%, AL-azo-H colloidal dispersions were obtained, and the average particle size of the formed spheres was approximately 170 nm. Li et al. [74] prepared lignin hollow microspheres from the esterified organosolv lignin modified with maleic anhydride using the self-assembly method in the mixed solvent of THF and water. Richter et al. [75] provided a simple self-assembly method for the synthesis of biodegradable lignin nanoparticles using organosolv lignin as a raw material. Specifically, organosolv lignin nanoparticles were obtained by gradually dropwise adding water into organosolv lignin/acetone solutions. Nanoparticles obtained from the above-mentioned preparation process showed a spherical shape and relatively uniform size (except organosolv lignin nanoparticles produced by Richter et al. (2016) due to the broad molecular weight distribution of organosolv lignin and inhomogeneous mixing, etc). However, there also are some limitations. They all utilized hazardous and expensive chemical reagents, such as acetyl bromide, cyclohexane, dioxane, NaNO_2_, maleic anhydride, THF, and acetone, or involved complicated chemical modification reactions. Li et al. [55] in their work, presented a simple, green, and low-cost preparation of nanocapsules through self-assembly from kraft lignin (KL) without any chemical reactions. During the process, water was added dropwise into the KL/ethanol solution by the peristaltic pump until the water content reached 90 vol%, at which the formation of KL nanocapsules was completed. The particle sizes of KL nanocapsules could be easily adjusted by changing the dropping speed of the water.

**Figure 2:**
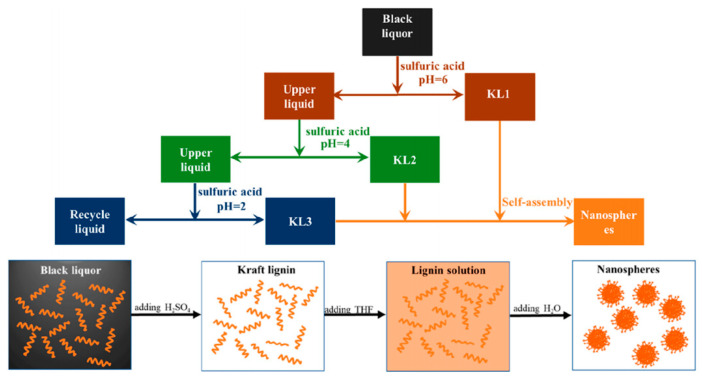
The synthetic route of lignin-based nanospheres [78]. Copyright © (2020) American Chemical Society.

**Figure 3:**
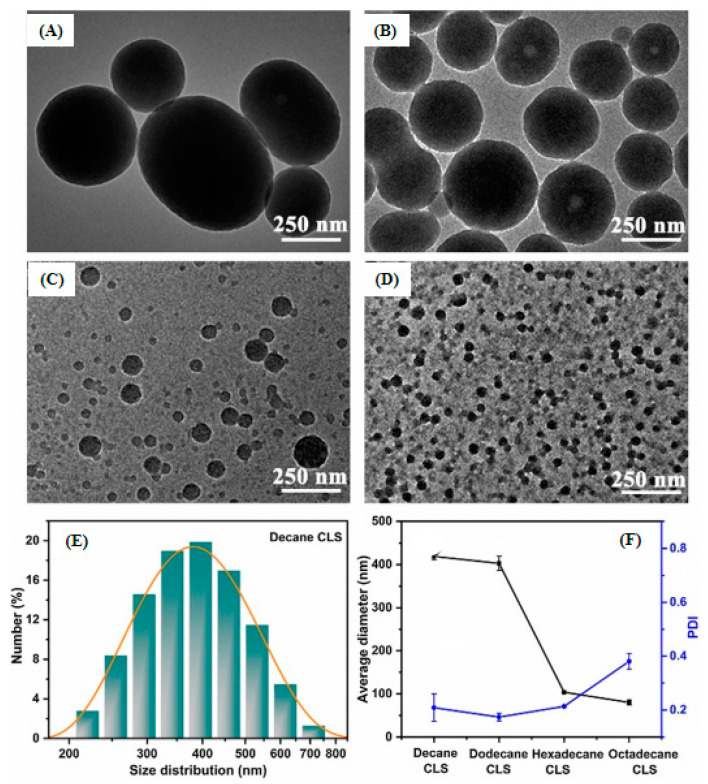
TEM images of CL spheres with various alkyl chains: (**A**) decane CL sub-micro spheres; (**B**) dodecane CL sub-micro spheres; (**C**) hexadecane CL sub-micro spheres; (**D**) octadecane CL sub-micro spheres; (**E**) size distribution of fresh decane CL sub-micro spheres; (**F**) effects of alkyl chains on PDI and diameter of alkylated CL sub-micro spheres [80]. Copyright © (2020) Elsevier.

Qian et al. [76] created an easy and practical self-assembly method to fabricate large, midsize, and small lignin-based colloidal spheres using the enzymatic hydrolysis lignin (EHL) and organosolv lignin (OL) as raw materials. Taking EHL as an example, large colloidal spheres were prepared by gradually adding NaCl aqueous solutions into the 10 g/L EHL acetone/water (8:1, vol/vol) solution. Midsize colloidal spheres were fabricated in much the same way, but NaCl aqueous solutions needed to be replaced with water. If small spheres were required, the concentration of EHL acetone/water (8:1, vol/vol) solution would be decreased to 0.1 g/L. Small nanoparticles were then formed by adding EHL solutions into water. Just by easily adjusting certain variables, lignin-based colloidal spheres with different sizes could be successfully obtained [74]. Huang et al. [77] separately used acetyl bromide, propionyl bromide, butyryl chloride, and valeryl chloride to perform hydrophobic modifications to AL in order to further understand the effect of the polarity and terminal alkyl chain length of acylation reagents on the lignin colloidal spheres. Then, the obtained four modified samples were prepared into colloidal spheres by the basically same process as created by Qian et al. [71]. Results showed that the formed acetylated lignin colloidal spheres exhibit a hollow spherical structure with only one hole, but the spheres gradually became porous configurations with the increase of alkyl chain lengths. Yan et al. [78] reported a facile self-assembly method to prepare size-controlled and super-term stable hollow or solid lignin-based nanospheres from kraft lignin. In this study, three different lignin samples labeled as KL1, KL2, and KL3 were first obtained by regulating the pH of black liquors to 6, 4, and 2 in the extraction, respectively. Then, water was uniformly added into the KL/THF solutions so as to prepare nanospheres (Figure 2). The results showed that the nanospheres formed exhibited a hollow spherical structure from KL1 and KL2 but a solid spherical configuration from KL3 and the size showed a decreasing trend from KL1 to KL3. This was caused by the decreased phenol hydroxyl content and the increased S/G ratio. Trevisan et al. [79] also separately produced lignin nanoparticles and acetylated lignin nanoparticles. The acid-alkali treatment was first performed on elephant grass to extract lignin for the acetylation and nanoparticle preparations. Then, a simple and easy self-assembly method was utilized to obtain nanoparticles in which water was poured into the acetone solution of lignin or acetylated lignin. In order to investigate the effect of hydrophobicity on particle size and stability, Li et al. [80] synthesized four different kinds of corncob lignin (CL) sub-micro spheres from four alkylated CL samples (decane alkylated CL, dodecane alkylated CL, hexadecane alkylated CL, and octadecane alkylated CL) with different hydrophobic properties but similar chemical structures in the mixed DMF/H_2_O system via self-assembly. The final results indicated that the particle size of spheres would decrease from 400 to 100 nm with the increasing length of n-alkane (Figure 3), and all spheres formed from the four alkylated CL samples exhibited an excellent dispersity in both water and acid-base environment.

All the above-mentioned works are focused on the nanoparticle preparation of alkali, kraft, organosolv, or enzymatic hydrolysis lignin. However, the investigation of the formation of the lignosulfonate nanoparticle is rare. As is well known, lignosulfonate has strong hydrophilicity, which makes it possible to transform lignosulfonate into reverse micelles. Zhong et al. [81] proposed an easy and green fabrication of sodium lignosulfonate reverse micelles (SLRMs). First, SL was dissolved into water to obtain aqueous SL solutions. Then, ethanol was gradually added, and the solutions started to become emulsified, thus forming SLRMs.

**Figure 4:**
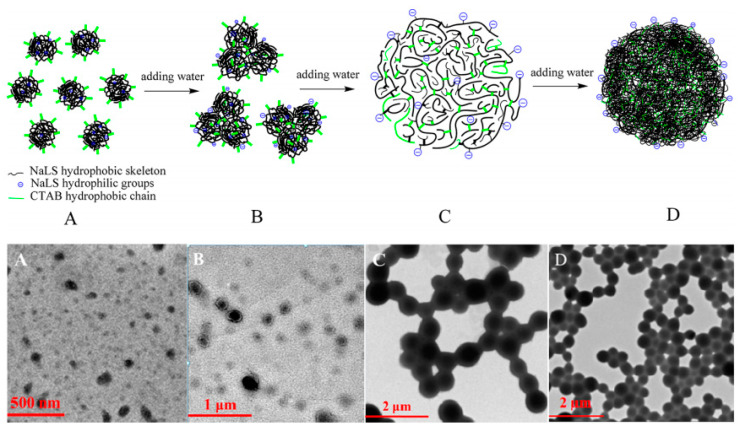
The schematic diagram of the colloid formation process of the lignosulfonate/CTAB complex system in mixing solvents of EtOH and H_2_O and the TEM images of samples formed from the dispersions with various water contents: (**A**) 0; (**B**) 0–58 vol %; (**C**) 58–84 vol %; (**D**) >84 vol % [7]. Copyright © (2018) American Chemical Society.

However, just due to this strong hydrophilicity, it is difficult for lignosulfonate to form normal micelles. To fill this gap, we presented a novel preparation method of lignosulfonate-based normal colloidal spheres via self-assembly in our previous work [7]. In this process, the cationic surfactant cetyltrimethylammonium bromide (CTAB) was first introduced to perform hydrophobic modifications to lignosulfonate molecules by a simple mixing method. Since lignosulfonate is a negatively charged anionic surfactant. The introduced CTAB would attach to the surface of lignosulfonate through electrostatic attractions, causing the shielding of the hydrophilic functional groups and hence increasing the hydrophobicity. Then, colloidal spheres were successfully prepared from the lignosulfonate/CTAB complex system at the stoichiometric mass ratio in the mixed solvent of ethanol and water through self-assembly (Figure 4). This whole preparation process was very simple, safe, and low-cost without any complicated chemical modification reactions, toxic or expensive chemical reagents. The formation of colloidal spheres from lignosulfonate not only provides a valuable and green approach to exploit the functionality of lignosulfonate and other technical lignin products but also gives some significant enlightenment on how to change the unordered and sophisticated lignosulfonate-based aggregates into ordered nanospheres. Soon afterward, other researchers in our group adopted a similar method to prepare lignin-based nanospheres. Instead of lignosulfonate, Li et al. [82] employed alkali lignin (AL) as raw materials. In this work, AL was first modified into cationic quaternized alkali lignin (QAL) by the quaternization reaction. Then, anionic sodium dodecyl benzenesulfonate (SDBS) was added and colloidal spheres from the SDBS/QAL complex were successfully obtained in the mixed ethanol/water solvent through self-assembly.

### 3.2. Solvent Exchange Method

A straightforward solvent exchange method was developed by Lievonen et al. [83] so as to prepare nanoparticles from waste lignin extracted from the kraft pulping process. In this study, the lignin was first dissolved into THF, and then the obtained lignin/THF solutions were placed into a dialysis bag, which was subsequently immersed in excess water. The lignin nanoparticles were formed after the dialysis process continued for at least 24 h. This whole preparation process was very simple which did not involve any chemical modification reactions. The size of nanoparticles could be adjusted by changing the pre-dialysis concentration of lignin/THF solutions. The obtained nanoparticles had excellent stability in the pure water at room temperature but tended to aggregate at a very high salt concentration or low pH. Lintinen et al. [84] synthesized metal-organic nanoparticles from the iron isopropoxide treated softwood kraft lignin. In this method, Fe(OiPr)_3_/THF or Fe(OiPr)_2_/THF solutions and lignin/THF solutions were first prepared, and then the solution of Fe:lignin in THF was obtained by adding THF solutions of Fe(OiPr)_3_ or Fe(OiPr)_2_ or a mixture of both (2:1) into THF solutions of lignin. After hydrolysis reactions, the whole system was moved into a dialysis tube immersed in water for the solvent change. Finally, the metal-organic nanoparticles were successfully synthesized without any further purification. This work gave a simple method of preparing different metal-organic nanomaterials. Based on Lievonen et al. [83] and Lintinen et al. [84], Figueiredo et al. [85] prepared pure lignin nanoparticles and iron(III)-complexed lignin nanoparticles. Fe_3_O_4_-infused lignin nanoparticles were prepared by mixing an equal mass of THF solutions of lignin and THF solutions of oleic acid-coated Fe_3_O_4_ nanoparticles followed by water dialysis. Zikeli et al. [86] also prepared nanoparticles from lignin separated from wood wastes according to Lievonen et al. [83] by first dissolving lignin into DMSO and then performing dialysis with an excess of water.

### 3.3. Acid Precipitation Method

**Figure 5:**
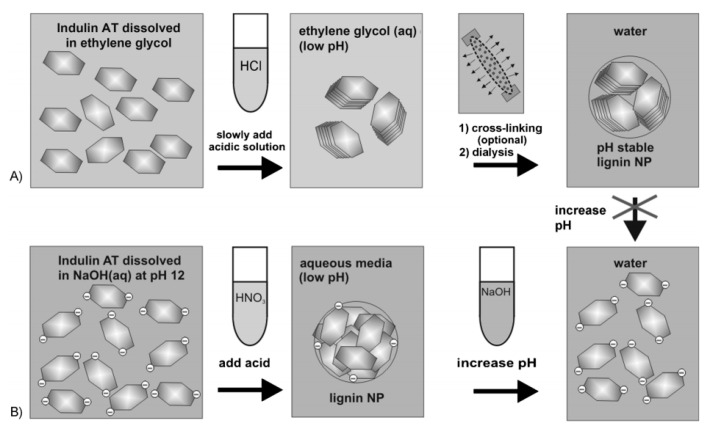
The proposed formation mechanism of lignin nanoparticles obtained through (**A**) precipitation of Indulin AT from the ethylene glycol solutions with HCl solutions followed by cross-linking and dialysis, and through (**B**) precipitation of Indulin AT from the aqueous solutions with high pH to low pH [87]. Copyright © (2012) John Wiley and Sons.

Frangville et al. [87] developed a unique acid preparation method for the fabrication of lignin nanoparticles. In this work, two alternative approaches were provided. In the first method, the lignin nanoparticles were obtained in the form of precipitates by adding hydrochloric acid into the ethylene glycol solution of lignin followed by cross-linking and water dialysis, which could keep stable at a wide range of pH. In the second method, the lignin nanoparticles were formed by rapidly adding HNO_3_ solutions into the high pH aqueous lignin solutions, which was stable only at low pH (Figure 5). Gupta et al. [88] used a similar acid precipitation method to Frangville et al. [87] to prepare lignin nanoparticles, which were then characterized by means DLS and TEM. Pang et al. [89] also synthesized nanoparticles from alkaline lignin with the acid precipitation method as discussed above. First, the alkaline lignin was dissolved in water and the pH was adjusted to 11 using NaOH solutions. Then, lignin nanoparticles were obtained by adding HCl to decrease the pH to 2.5 under stirring.

Another unique acid precipitation method to prepare three different kinds of lignin nanoparticles was developed by Rahman et al. [90] using castor oil, ethylene glycol, and water as solvents, respectively. The results showed that the obtained nanoparticles had a particle size in the range of 15–20 nm and exhibited an obvious spherical morphology when ethylene glycol and castor oil were employed as solvents.

### 3.4. Polymerization Method

Early in 2006, Barakat et al. [91] in their work reported the synthesis of nanoparticles from arabinoxylan-dehydrogenation polymer (a synthetic lignin polymer) through the polymerization method. In this process, coniferyl alcohol, and sinapyl alcohol were polymerized in the presence of heteroxylans to prepare the arabinoxylan-dehydrogenation polymer nanoparticles. Then, the morphology of the produced nanoparticles was characterized by means of TEM and multidetected size exclusion chromatography.

Another novel method for the synthesis of lignin-based nanoparticles was developed by Qian et al. [92] by grafting 2-(diethyl-amino)ethyl methacrylate (DEAEMA) to alkali lignin via atom transfer radical polymerization (ATRP). The resulting nanoparticles could be employed as surfactants for CO_2_/N_2_-switchable Pickering emulsions, which represented an innovative approach in developing a high value-added application of lignin products.

### 3.5. Ultrasonication Method

Despite the disadvantages of broad size distributions, the ultrasonication method as well as other mechanical treatments are still widely utilized to decrease the particle size to the nanometer scale due to its simpleness and facility. Gilca et al. [93] prepared lignin nanoparticles by sonicating the aqueous lignin suspensions for 60 min followed by being dried under mild conditions. In order to evaluate the structural and compositional variation before and after sonication, two different types of nanoparticles were prepared from wheat straw lignin and Sarkanda grass lignin, respectively. Results indicated the nanoparticles obtained in the above-mentioned two cases both had a particle size in the range of 10 to 50 nm in spite of the not very regular shape, which showed an obvious decrease compared to the raw lignin. Moreover, the applied ultrasonication intensity would not cause significant structural and compositional variations of nanoparticles. Zhou et al. [94] also prepared alkali lignin and alkali lignin/polydopamine-based nanocapsules via ultrasonication. In contrast to nanoparticles obtained by Gilca et al. [93], the resulting capsules in this work all exhibited a relatively regular spherical structure.

### 3.6. Crosslinking Method

Yiamsawas et al. [95] first synthesized the biodegradable hollow nanocontainers with a hydrophilic core from sodium lignosulfonate and alkali lignin. For the preparation of lignosulfonate nanocontainers, sodium lignosulfonate was first dissolved in aqueous NaCl solutions to generate the dispersed phase, which was subsequently mixed with cyclohexane containing the biocompatible surfactant PGPR (poly-glycerol polyricinoleate). The obtained pre-emulsion was then ultrasonicated so as to form a stable mini-emulsion. The polyaddition reaction occurred at the interface of the mini-emulsion droplets, which was initiated by dropwise adding toluene diisocyanate (TDI)/cyclohexane solutions into the mini-emulsion. After keeping at room temperature overnight, the lignosulfonate nanocapsule dispersions were successfully formed, which could still remain stable when being transferred into aqueous dispersions because of the presence of sulfonic groups. The preparation process of alkali lignin nanocapsules is basically the same, but sodium dodecyl sulfate should be added if the nanocapsules are required to redisperse in water. These obtained lignin nanocontainers had a particle size in the range of 150–200 nm and could keep stable in aqueous or organic dispersions over a long period (several weeks or even months). Tortora et al. [96] created a novel synthesis of kraft lignin microcapsules by first preparing oil in water emulsions followed by ultrasound-assisted crosslinking of lignin at the oil/water interface. Taking the ultrasound preparations of lignin microcapsules in the presence of H_2_O_2_ as an example, the specific process was described as follows. Firstly, olive oil and H_2_O_2_ were added into lignin alkali solutions, and then the whole mixing system was sonicated. Next, lignin microcapsules were obtained by centrifuging and washing. The finally formed lignin microcapsules had an average particle size of 0.3–1.1 μm with a spherical configuration. The formation mechanism was also revealed by means of GPC and NMR measurements. Li et al. [97] fabricated porous lignosulfonate spheres from the cross-linking reaction of lignosulfonate and sodium alginate using epichlorohydrin as a cross-linking agent followed by dropwise adding into CaCl_2_ solutions for gelation and solidification. Results showed that the formed lignosulfonate spheres exhibited an obvious porous structure with a large pore volume and a high porosity. Nypelö et al. [98] synthesized lignin supracolloids by first adding lignin alkali solutions into octane containing surfactant mixtures of Span 80, Tween 80, and 1-pentanol to prepare microemulsions, and then adding epichlorohydrin into microemulsions. In another research carried out by Chen et al. [99], pH-responsive lignin-based nanocapsules were successfully prepared by an interfacial mini-emulsion polymerization. In the first step, the water phase containing lignosulfonate and water without or with SDS was mixed with the oil phase containing butyl acetate, hexadecane (co-stabilizer), AIBN (an oil-soluble initiator), and trimethylolpropane tris(3-mercapto propionate) (a cross-linker), which was followed by sonication. In the second step, interfacial mini-emulsion polymerization was performed in the obtained mini-emulsion system to form nanocapsules. This work provided a facile method to make use of waste biomaterials from biorefinery industries.

### 3.7. CO_2_ Antisolvent Method

CO_2_ as an antisolvent to produce polymeric nanoparticles has attracted special interest due to advantages such as abundance, low costs, nontoxicity, nonflammability, and poor solubility for macromolecules [100]. Lu et al. [101] prepared nanoscale lignin from non-nanoscale lignin by means of the supercritical antisolvent (SAS) process. First, acetone was pressed into a precipitation chamber filled with SC-CO_2_ through the liquid pump in order to obtain stable precipitation reaction conditions. The acetone was stopped once the stable conditions were achieved. Then, the lignin/acetone solution was delivered into the precipitation chamber through a stainless-steel nozzle. Once the delivery was completed, the liquid pump was stopped, but the SC-CO_2_ continued to keep flowing to remove the residual organic solvents. Finally, the nano-scale lignin was obtained and could be taken out as the pressure of the precipitation chamber decreased to atmospheric pressure. Results indicated that the formed nanoscale lignin had an average particle size of about 0.144 μm, and there were no chemical changes when non-nanoscale lignin was converted into nanoscale lignin. Myint et al. [102] utilized a similar method to Lu et al. [101] to fabricate environmentally friendly nanoparticles from kraft lignin using DMF as an organic solvent. Then, they investigated the effect of different process parameters on the properties of the particles, revealed the formation mechanisms, and elucidated the quality of lignin nanoparticles by means of FESEM, HRTEM, BET, ATR-FTIR, XPS, XRD, DSC, TG/DTA, and UV-vis analyzers.

## 4. Industrial Application of Lignin-Based Nanoparticles

In recent years, lignin-based nanoparticles have been widely used as biomaterials for a large range of applications, such as drug delivery, UV absorbents, hybrid nanocomposites, and antioxidant agents. In the following section, we would summarize the most common applications of lignin-based nanoparticles.

### 4.1. Drug Delivery

Numerous researches have shown that lignin-based nanoparticles had the capacity of encapsulating different compounds for various pharmaceutical applications. Qian et al. [71] developed colloidal spheres from the acetylated lignin in the mixed solvent of THF and water by means of the self-assembly method, which showed potential applications in drug delivery and controlled release, and pesticide microencapsulation fields. Deng et al. [73] utilized the obtained hollow lignin azo colloids to encapsulate pesticide avermectin (AVM). Results showed that the hollow lignin azo colloidal spheres have a high AVM encapsulation efficiency which was determined to be 61.49% (*w*/*w*) by means of high-performance liquid chromatography measurements. The cumulative release amount of AVM after 120 h was 84% and the release process was still continuing, which indicated that the hollow lignin azo colloidal spheres exhibited excellent controlled release performance for AVM. Figueiredo et al. [85] fabricated three different kinds of lignin nanoparticles pLNPs, Fe-LNPs, and Fe_3_O_4_-LNPs with low cytotoxicity. For pLNPs, they could effectively loaded some drugs or other cytotoxic agents with poor water solubility such as sorafenib (SFN) and benzazulene (BZL), and also improved their release profiles at different pH values in a sustained manner. Moreover, the obtained BZL-pLNPs showed an enhanced antiproliferation effect in contrast to the pure BZL. Dai et al. [103] prepared magnetic resveratrol (RSV) loaded lignin nanoparticles (AL/RSV/Fe_3_O_4_ NPs) by dropwise adding Fe_3_O_4_ nanoparticle solutions to the methanol (ethanol or THF) solutions of alkali lignin and RSV. These obtained AL/RSV/Fe_3_O_4_ NPs could be used as a novel and highly-efficient nano delivery, which showed a good anti-cancer effect and could obviously improve the in vitro RSV release and stability, accumulation, and anticancer efficacy comparing to free drugs. Li et al. [104] synthesized lignosulfonate-based colloidal spheres from the mixture of sodium lignosulfonate (SL) and CTAB through self-assembly for the encapsulation of photosensitive AVM. The obtained AVM@SL-CTAB microencapsulation had a uniform spherical shape, and its AVM encapsulation efficiency reached up to 62.58%. The controlled release experimental results showed that AVM@SL-CTAB was still continuing to release AVM after 70 h, and the cumulative release amount at 70 h was determined to be 49.96%. Furthermore, this release process of AVM@SL-CTAB microencapsulation could be adjusted by changing the addition of SL-CTAB colloidal spheres. In another study performed by Li et al. [105], pH-responsive complex micelles were synthesized from quaternized alkali lignin and sodium dodecyl benzenesulfonate (SDBS) in green solvents and used to encapsulate the oral drug Ibuprofen (IBU) through hydrophobic interactions. The encapsulation efficiency was calculated to be 74.44%. The in vitro release behavior indicated the formed microencapsulation exhibited excellent pH-dependent controlled release performance of IBU. This study presented a novel approach to fabricate oral drug delivery carriers, which is significant for the high value-added application of lignin. The same researches also employed the complex colloidal spheres to encapsulate pesticide AVM, which showed a remarkable encapsulation efficiency. The cumulative release amount of AVM from the microencapsulation was 77% after 72 h, and the release process was still going on. This release behavior of AVM encapsulated by colloidal spheres could be adjusted by controlling the mass ratio of colloidal spheres to AVM [82]. Mishra et al. [106] fabricated solid and hollow colloidal spheres from dioxane soluble fractions of alkali lignin using an ultrasonic spray-freezing route without any chemical modifications, which could be potentially used in drug delivery system. Chen et al. [107] developed a novel and facile approach to prepare lignin nanoparticles through self-assembly in aqueous sodium p-toluenesulfonate (pTsONa) solutions. Due to hydrotropism, these formed nanoparticles could dissolve and encapsulate various water-insoluble or water-soluble drugs, and the encapsulation efficiency can reach up to 90%. Additionally, the lignin nanoparticles could also achieve the sustained release of different drugs.

### 4.2. UV Absorbents

**Figure 6:**
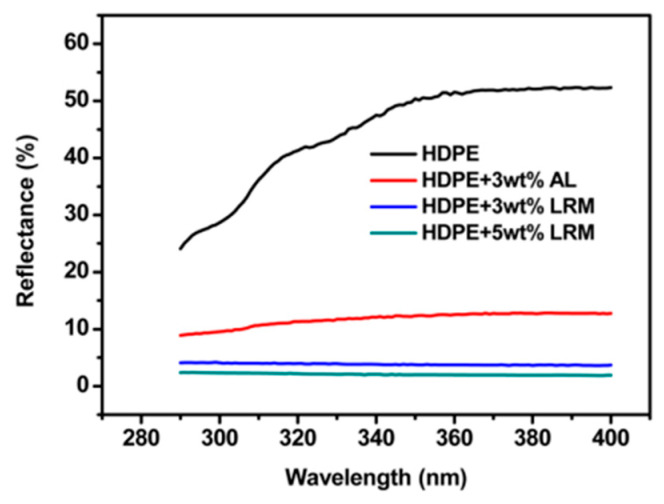
UV reflectance of HDPE blended with various amounts of AL and LRM [72]. Copyright © (2015) American Chemical Society.

Lignin has huge potential as UV absorbents because of its excellent oxidation resistance. Qian et al. [72] prepared lignin reverse micelles (LRMs) via self-assembly. When lignin was transformed into reverse colloidal spheres, the hydrophobicity was greatly improved, which resulted in the enhanced miscibility of LRMs and high-density polyethylene (HDPE). Because LRM still retained the phenolic hydroxyl of lignin, the obtained HDPE/LRM composite materials exhibited excellent UV-absorbing properties (Figure 6). The same researches also fabricated three different sizes (large, midsize, and small) of normal lignin colloidal spheres by the self-assembly method and blended them with pure skin creams in order to develop lignin-based sunscreens. The results indicated that the sunscreen performance of creams with lignin colloidal spheres was improved compared to that with original lignin, and the size of colloidal spheres would influence the sunscreen performance, which showed a decreasing tendency with the increasing sizes of colloidal spheres. For the sun-blocking process, phenolic hydroxyl groups played a crucial role. If lignin was acetylated to shield the phenolic hydroxyl groups, SPF values would decrease dramatically. On the contrary, when the phenolic hydroxyl group content increased, the SPF values would increase. For example, when 10 wt % small size organosolv lignin colloidal spheres with more phenolic hydroxyl groups were added, the SPF value reached up to 15.03. This study gave an easy method to fabricate natural lignin-based sunscreens with high UV-blocking properties [76].

Zikeli et al. [86] utilized synthesized lignin nanoparticles to perform wood surface treatment by the dip-coating technique. Results showed that nanoparticles from lignin extracted from Iroko or Iroko-Norway spruce mixed sawdust could provide better UV resistance to wood due to the presence of aromatic extractive compounds in Iroko lignin macromolecules. In another research carried out by Wang et al. [108], regular lignin nanoparticles with high yields were prepared. In this method, lignin was first modified by a microwave acetylation process, and then lignin nanoparticles were fabricated through a solvent exchange combined ultrasound process. When the obtained lignin nanoparticles were added into chemical cream, the sunscreen performance was improved, and the sunscreen performance of small nanoparticles was better than that of large nanoparticles. Ju et al. [109] developed a simple continuous method to synthesize lignin nanoparticles in a microchannel reactor via a liquid precipitation process using polyvinylpyrrolidone (PVP) and sodium dodecyl sulfate (SDS) as stabilizers. When these resulting nanoparticles were added into poly(vinyl alcohol) (PVA) films, the UV-shielding efficacy was enhanced by 13.3% (in the ultraviolet spectrum of 250 nm) in comparison to PVA films with raw lignin. Trevisan et al. [79] incorporated the obtained lignin nanoparticles from elephant grass into a neutral cream. The UV-vis light-shielding performance of the blended creams with lignin nanoparticles was enhanced compared with commercial sunscreens with FPS 30, and it increased with the increasing percentage of nanoparticles in the cream. Zhou et al. [94] first synthesized polydopamine-grafted lignin (AL-PDA) through the free radical addition of alkali lignin (AL) and dopamine (DA). Then, they employed the formed AL-PDA to emulsify organic UV filters followed by further cross-linking to prepare nanocapsules through ultrasonic cavitation. When these obtained bioinspired AL-PDA nanocapsules with strong bioadhesion were used as an ingredient (with a dosage 10 wt %) for formulating sunscreen, the sun protection factor value can reach 195.33 lasting for more than 8 h under UV radiations. Due to the excellent antioxidant capacity and biocompatibility, the as-prepared nanocapsules showed a superior performance and could be used securely in the sunscreen.

### 4.3. Hybrid Nanocomposites

Lignin nanoparticles are often blended with polymers as reinforcing agents, which make the obtained copolymers exhibit better mechanical, thermal, and biocompatible properties than the original polymers. Chung et al. [110] transformed lignin into a lignin-g-poly(lactic acid) (PLA) copolymer by grafting lactide onto lignin using triazabicyclodecene (TBD) as a catalyst so as to improve its miscibility with other bioplastics. The chain length of PLA could be adjusted by preacetylation treatments or changing the ratio of lignin and lactide. When 10% lignin-g-PLA copolymers were added, both the UV absorbance and mechanical properties of PLA composites improved. Jiang et al. [111] prepared nano-lignin from lignin and poly (diallyl dimethylammonium chloride) (PDADMAC) complexes (LPCs) through self-assembly. The obtained lignin particles had an average size of less than 100 nm and could stably disperse in aqueous solutions. When the nano-lignin was blended with natural rubber (NR) latex, it could be homogeneously dispersed in the NR latex at the nanoscale, which resulted in the improved thermal stability and mechanical properties of LPCs/NR composites. Nair et al. [112] presented a novel method to convert large micron- to nano-sized kraft lignin particles to nanolignin particles using a simple high shear homogenizer, which would not cause any chemical composition changes. When these nanolignin particles were blended with polyvinyl alcohol (PVA), the thermal stability of obtained nanolignin/PVA composites increased in comparison with the original lignin/PVA composites. Qian et al. [72] introduced the formed lignin reverse micelles (LRMs) into high-density polyethylene (HDPE). Taking the LRM addition of 5 wt % as an example, it was observed that the elongation at break increased to 1030% from 671% and Young’s modulus increased to 2104 MPa from 1066 MPa, respectively. This indicated that the incorporation of LRMs could cause an obvious improvement in the mechanical properties of HDPE (elongation at break and Young’s modulus).

Gupta et al. [88] first synthesized lignin nanoparticles (LNP) by uniformly adding hydrochloric acid into the ethylene glycol solutions of lignin followed by dialysis in excess water, precipitation at pH of 2 with HCl solutions, centrifugation, washing with water, and drying. Then, 1.5 wt % of resulting products were added into bio-poly(trimethylene terephthalate) (bio-PTT) and the results showed that the tensile strength, modulus, flexural strength, modulus, and impact strength separately increased by 14.86%, 8.26%, 14.89%, 10.24%, and 30.93%, respectively, and the heat deflection temperature (HDT) increased by 117.7% comparing to the original bio-PTT matrix, indicating a good effect of LNP on improving the mechanical properties and thermal resistance of bio-PTT. Moreover, the obtained bio-PTT/LNP composites exhibited the highest water absorption ability. To improve the miscibility of lignin with plastics, Kai et al. [113] synthesized poly(methyl methacrylate) (PMMA) grafted lignin (lignin-PMMA) copolymers via atom transfer radical polymerization. These obtained copolymers were further added into poly(e-caprolactone) (PCL) and then engineered into nanofibrous composites through electrospinning. Tensile and dynamic rheological measurements and cell culture study displayed that the introduction of lignin-PMMA could cause a significant improvement of Young’s Modulus, tensile strength, and storage modulus, and the obtained nanofibrous composites showed good biocompatibility. Similar to Kai et al. [113], Yang et al. [114] synthesized Poly(methyl methacrylate) grafted lignin nanoparticles (PMMA-g-LNP) copolymer by solvent-free radical polymerization and blended these resulting copolymers into the commercial PMMA. Results indicated that the obtained PMMA/LNP nanocomposites exhibited improved hardness values, UV resistance, thermal and scratch resistance compared to the original PMMA matrix.

### 4.4. Antioxidant Agents

**Figure 7:**
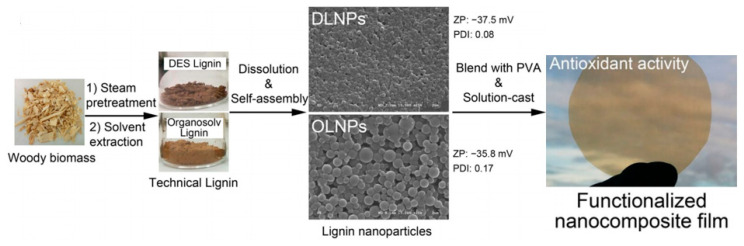
Preparation steps of lignin nanoparticles and lignin nanoparticles/PVA composite film [118]. Copyright © (2017) Springer Nature.

The functional groups such as methoxy and phenolic hydroxyl groups in lignin are able to cause the termination of oxidative propagation reactions through hydrogen donation [5,19]. Therefore, lignin nanoparticles could be introduced into various materials to produce antioxidant products with different applications. Lu et al. [101] developed nanoscale lignin through the supercritical antisolvent (SAS) method employing supercritical carbon dioxide as the antisolvent and acetone as the solvent. Due to the increased solubility in water, the resulting nanoscale lignin had a higher antioxidant activity, which exhibited enhanced superoxide radical scavenging activity, DPPH radical scavenging activity, and reducing power. In another study performed by Ge et al. [115], nanolignin was prepared through the alkaline solution precipitation method. Free Radical Scavenging (FRS) activity analysis displayed that nanolignin showed a 3.3-fold higher activity in contrast to the control sample. According to the 2,2-Diphenyl-1-picrylhydrazyl (DPPH) antioxidant assay, the IC50 value increased to 2.70 ± 0.17 mg/mL for nanoscale lignin from 32.21 ± 0.1 mg/mL for microscale lignin, suggesting a higher antioxidant activity of nanoscale lignin. Yang et al. [116] introduced the obtained lignin nanoparticles (LNP) via acid precipitation into a poly(vinyl alcohol) (PVA)/chitosan (CH) mixture. DPPH radical scavenging activity measurement results showed that LNP exhibited a synergic effect with CH in antioxidant responses of the resulting PVA/CH/LNP nanocomposites. Together with the antimicrobial activity, PVA/CH/LNP nanocomposites could be potential candidates for use in various biomedical applications such as tissue engineering, drug delivery, and wound healing. Yearla et al. [117] fabricated two different kinds of alkali lignin nanoparticles (ALNP) and dioxane lignin nanoparticles (DLNP). Compared to the original alkali lignin and dioxane lignin polymers, both ALNP and DLNP showed higher antioxidant activity according to the radical scavenging activity analysis. Additionally, DLNP could provide more pronounced protection for *Escherichia coli* against UV than ALNP. On account of these good antioxidant and UV protection properties, DLNP can be therefore further applied in pharmaceutical, food, and cosmetic industries. Tian et al. [118] prepared two types of LNP via self-assembly from ethanol-organosolv and deep eutectic solvent (DES) extracted lignins, respectively. When these lignin nanoparticles were incorporated into PVA, the resulting copolymer obtained additional antioxidant functionalities (Figure 7). He et al. [119] firstly produced LNP through an acid precipitation method, which was then esterified and etherified by citric acid to form modified LNP (MLNP). When LNP and MLNP were also separately blended with PVA through the solvent casting approach, the antioxidant property of the obtained MLNP based nanocomposite films was superior to that of LNP based nanocomposite films, which provided potential for these materials as environmental friendly antioxidant additives in the food and packaging industry.

### 4.5. Other Applications

In addition to the above, lignin-based nanoparticles have also been exploited for other industrial applications, such as antibacterial agents, adsorbents to remove heavy metal ions, and dyes as well as anticorrosive nanofillers.

The presence of phenolic components endows an antimicrobial characteristic to lignin, which therefore could be utilized as an antimicrobial agent. Kim et al. [120] developed nanoparticles combining chitosan and lignosulfonates for the first time. When lignosulfonate was incorporated into chitosan nanoparticles, the obtained composite nanoparticles showed greater antimicrobial activity in comparison with chitosan nanoparticles alone.

Additionally, lignin could also act as an adsorbent to remove heavy metal ions and dyes. Li et al. [97] utilized the obtained porous lignosulfonate spheres (PLS) through a feasible gelation-solidification method, which possessed a total pore volume of 0.416 cm^3^/g and high porosity of 87.66%, to adsorb lead ions. The adsorption experiments showed that the adsorption efficiency of PLS for lead ions reached up to about 95.6% at the initial concentration of 25.0 mg/L, suggesting an excellent adsorption capacity of PLS. Therefore, PLS could be applied for the continuous treatment of industrial wastewater rich in heavy metals. Azimvand et al. [121] studied the removal of Safranin-O from aqueous solutions using lignin nanoparticles and lignin nanoparticle-g-polyacrylic acid as adsorbents, respectively. In this work, lignin nanoparticles (LN) were fabricated by first dissolving the alkali lignin (AL) in polyethylene glycol and then slowly adding hydrochloric acid into AL/polyethylene glycol solutions, and lignin nanoparticle-g-polyacrylic acid (LN-g-PAA) was prepared through the copolymerization reactions between polyacrylic acid and lignin nanoparticle using potassium persulfate as a radical initiator. Results indicated the adsorption capacity of LN-g-PAA copolymer increased nearly 1.4-fold in LN. Therefore, LN-g-PAA was considered to be proper for removing Safranin-O dye from wastewater.

Ultimately, Rahman et al. [90] reported the use of lignin nanoparticles as anticorrosive nanofillers. In this research, they evaluated the anticorrosive behavior of three kinds of lignin nanoparticles (LNP) via acid precipitation in different mediums (castor oil, ethylene glycol, and water). Results showed that all three kinds of lignin nanoparticles could be dispersed in the epoxy matrix-forming nanocomposite coatings to protect the carbon steel (CS) from corrosion in a highly saline environment, Moreover, the LNP-dispersed epoxy coatings could provide better protection compared to the bare epoxy coating.

## 5. Conclusions

In spite of the increasing utilization of lignin-based biomaterials, the high value-added applications of lignin still face some challenges mainly due to its complicated and changeable macromolecular structure. One way to deal with this problem is to convert unordered raw lignin into uniform nanoparticles, which could be promising candidates for various applications. Unfortunately, most preparation methods for lignin-based nanoparticles use expensive or environmentally hazardous solvents including THF, DMSO, and DMF, which are expected to be replaced with cheap and green solvents such as water and ethanol. Meanwhile, the yields of some processes to obtain nanoparticles are relatively low. Therefore, the increase of yields is necessary from both economic and application points of view. In the future, we hope to create preferable preparation methods for lignin-based nanoparticles which are more low-cost, eco-friendly, and easier for large-scale production. Additionally, in order to further broaden the application performance, some special functional groups could be introduced to improve the thermal stability, conductivity, adsorption, magnetic and optical properties, etc. of lignin-based nanoparticles.

## Figures and Tables

**Table 1:** Treatment conditions [5], solubility, chemical compositions [19], advantages and disadvantages of technical lignin (ND—No data). Copyright © (2018) Elsevier; Copyright © (2019) John Wiley and Sons.

Parameter	Lignosulfonate	Kraft Lignin	Organosolv Lignin	Soda Lignin	Enzymatic Hydrolysis Lignin
Treatment conditions	Metal sulfite + sulfur dioxide (Ca^2+^, Mg^2+^ or Na^+^) (pH = 2–12, T = 120–180 °C, for 1–5 h)	First: Sodium hydroxide and sodium sulfide (pH = 13–14, T ≈ 170 °C); Second: Sulfuric acid (pH = 5–7.5)	Organic solvents (such as acetone, ethanol, and methanol), usually mixed with water (T = 170–190 °C)	13–16 wt % of sodium hydroxide solutions (T = 140–170 °C) + anthraquinone (catalyzer)	Cellulases and hemicellulases [10]
Solubility	Water	Alkali, organic solvents	Organic solvents	Alkali	Partially in organic solvents
Ash content (mass%)	4.0–9.3	0.5–3.0	1.7	0.7–2.3	1.0–3.0
Sulfur (%)	3.5–8.0	1.0–3.0	0	0	0–1.0
Carbohydrates (mass%)	ND	1.0–2.3	1–3	1.5–3.0	10.0–22.4
Molecular weight (Da)	1000–50,000	1500–5000	500–5000	1000–3000	5000–10,000
PDI	4.2–8.0	2.5–3.5	1.5–2.5	2.5–3.5	4.0–11.0
Advantages	A good aqueous solubility [5]	A higher purity [41]	A higher purity [9]; sulfur-free; basically preserves the native structure of lignin [5]	Sulfur-free [41]	Basically non-sulfur; possesses a closer structure to native lignin [19]
Disadvantages	A structural change of lignin and the low purity after isolation [5]	A worse solubility [19]	Difficult to produce on a large scale [1]	Difficult to recover through centrifugation or filtration [56]	Very low solubility in either water or some organic solvents [19]
